# Resolution of an unexpected ABO typing discrepancy in a 9‐month‐old patient with juvenile myelomonocytic leukemia

**DOI:** 10.1002/ccr3.3101

**Published:** 2020-07-15

**Authors:** Daniela Hermelin, Danmei Zhang, Douglas Blackall

**Affiliations:** ^1^ Department of Pathology Saint Louis University School of Medicine Saint Louis Missouri USA; ^2^ Department of Pathology SSM Health Cardinal Glennon Children’s Hospital Saint Louis Missouri USA

**Keywords:** ABO typing discrepancy, leukemia, stem cell transplant

## Abstract

In the post–hematopoietic transplant period, the components of the ABO blood type (antigen testing of erythrocytes and plasma antibody testing) can provide important insights into a patient's immunologic status.

## Graphical abstract

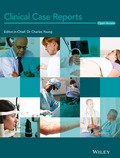

## 1 INTRODUCTION

ABO typing discrepancies are relatively uncommon occurrences in the hospital transfusion service but are routinely encountered in the setting of hematopoietic progenitor cell transplantation (HPCT).^1^ We were challenged recently with a typing discrepancy that occurred in a 9‐month‐old male patient who underwent an unrelated allogeneic umbilical cord blood transplant for juvenile myelomonocytic leukemia. The discrepancy was resolved but shed light on the value of utilizing the simple, yet informative ABO type to monitor HPCT patients in order to provide feedback on engraftment status and the immunologic resetting that occurs post‐transplantation.

## 2 CASE PRESENTATION

The original blood type of the patient was O‐positive with an anti‐A titer of 8 and an anti‐B titer of 4. The donor was AB‐positive. The patient received a conditioning regimen consisting of busulfan, cyclophosphamide, melphalan, and rabbit antithymocyte globulin. The total number of transfused CD34 + cells was 1.76 × 10^6^/kg. Graft‐versus‐host disease prophylaxis consisted of tacrolimus and mycophenolate mofetil. The hematology‐oncology service set transfusion parameters as follows: red blood cells (RBCs) for a hemoglobin < 6.5 mg/dL (without symptoms) or <8 mg/dL (with symptoms) and platelets for a platelet count < 20 000/µL (without bleeding) or <30 000/µL (with minor bleeding). During the first 60 days after transplant, the patient was heavily transfused receiving a total of 38 AB platelet products and 19 group O RBCs. These units were all irradiated and CMV‐seronegative. Neutrophil engraftment, defined by an absolute neutrophil count > 500/µL, was achieved on Day + 18. Platelet engraftment, defined by an untransfused platelet count > 20 000/µL, was achieved on Day + 55.

ABO typing, both forward (antigen on cells) and reverse (antibody in plasma), and Rh testing were performed every 3 days using a gel method. On Day + 25, the forward type demonstrated the evidence of conversion from O to AB, as the anti‐A and anti‐B reagents were weakly reactive with a mixed‐field pattern. The reverse type continued to demonstrate anti‐A but was negative for anti‐B. However, on Day + 50, neither anti‐A nor anti‐B was detected. These findings were consistent for the next three determinations. Unexpectedly, on Day + 66, an ABO typing discrepancy was again detected. The forward type remained AB, but the reverse type now demonstrated reactivity with group A reagent RBCs. We surmised that this unexpected result could represent residual native anti‐A, perhaps related to a relapse of the patient's disease, though this seemed unlikely as he was doing well clinically and his chimerism studies demonstrated full engraftment of female transplanted cells. We also investigated the possibility of a cold‐reactive antibody by incubating group O screening cells at both 37° and room temperature, but both results were negative. Additional testing (negative RBC reactivity with the anti‐A_1_ lectin, *Dolichos biflorus*) proved that the patient was now a subgroup of A and had developed anti‐A_1_, a relatively common occurrence in AB patients.^2^ This was confirmed by demonstrating negative plasma testing with A_2_ RBCs.

## 3 DISCUSSION

The ABO blood group system is the most important RBC antigen system in transfusion medicine.^2^ The primary antigens in this system, A, B, and H, are carbohydrates. In addition to their representation on RBCs, they are readily identified in other tissues, the gut epithelium being a particularly rich site of expression.^3^ In general, carbohydrate antigens engender an IgM immune response with greatest reactivity at colder temperatures (eg, 4°C); however, anti‐A and anti‐B are potent agglutinins at 37°C, which serves as the basis for the requirement to transfuse ABO‐compatible units of blood.^4^ In addition to their expression on human tissues, including RBCs, the A, B, and H antigens are present in nature.^3^ This exposure is the basis for the production of “naturally occurring” antibodies as an expected accompaniment of an individual's RBC antigen type (eg, anti‐B in those of the A blood group).

The A blood group antigen has multiple structural forms.^2^ The most common of these, which is designated A_1_, is found in approximately 80% of those individuals who react with anti‐A typing sera (ie, group A and group AB individuals). The next most common type, designated A_2_, is found in the vast majority of the remaining individuals. However, the A_2_ antigen is structurally different than the A_1_ antigen (it is less complex).^2^ In addition, there are fewer copies of the A_2_ antigen on RBCs in comparison with the A_1_ antigen.^4^ Thus, there are both qualitative and quantitative differences between A_2_ and A_1_. Interestingly, individuals of the A_2_ phenotype can generate antibodies with specificity for the A_1_ antigen.^2^ These are naturally occurring immune responses but are rarely clinically significant as they are largely cold‐reactive. It was the generation of an anti‐A_1_ immune response in our patient, as he was converting to the AB blood group (specifically A_2_B) that led to the unexpected ABO typing discrepancy described in this paper. Although such discrepancies are routinely seen in the peri‐transplant period, when patients are in the process of engrafting and changing their native blood type, our patient had engrafted successfully and then presented with an unexpected ABO typing discrepancy. A thorough serological investigation revealed that this newly “minted” AB patient was actually A_2_B and had mounted an A_1_ immune response.

One of the most basic yet important tools in the blood bank is the ABO blood type. In the peri‐transplant time period, the strength and evolution of the forward type can determine red blood cell engraftment status and may predict disease relapse.^5^ In contrast, establishing a concordant reverse type correlates with a resetting of the patient's immune system in which immune tolerance is reestablished after transplant.^6^ Although the specific mechanisms driving this process are largely unknown, it likely involves central tolerization of precursor B cells upon exposure to the newly engrafted erythroid compartment.^7^ In sum, two discrete processes can be monitored with ABO typing as a routine part of HPCT patient care.

In our case, a typing discrepancy involving a change in the patient's reverse type, occurring on Day + 66 of an allogeneic cord blood transplant, led to a blood bank investigation that revealed an AB donor with a subgroup of A. Although this resulted in neither patient care nor transfusion support issues, it shed light on the importance of using the ABO type to monitor engraftment and immunologic status during HPCT. In addition to providing therapeutic support for transplant patients during the critical engraftment period, the blood bank can also provide insight into a patient's immunologic status and guide clinical colleagues accordingly.

## CONFLICT OF INTEREST

The authors have no conflicts of interest to declare.

## AUTHOR CONTRIBUTIONS

DH: evaluated data and wrote the original manuscript. DZ: collected and analyzed the data. DB: conceived the manuscript idea, evaluated the data, and revised the manuscript.

## ETHICAL APPROVAL

Not applicable.
